# Developmental stage–dependent effects of cerebrospinal fluid on hair follicle stem cell proliferation, morphology, and neural-associated gene expression

**DOI:** 10.1016/j.ibneur.2026.09.015

**Published:** 2026-09-23

**Authors:** Sareh Pandamooz, Zahra Tavakkoli, Jaleel A. Miyan, Mohammad Saied Salehi

**Affiliations:** a Department of Biology, Faculty of Sciences, Shiraz University, Shiraz, Iran; b Stem Cells Technology Research Center, Shiraz University of Medical Sciences, Shiraz, Iran; c Faculty of Biology, Medicine & Health, Division of Neuroscience & Experimental Psychology, The University of Manchester, Manchester, UK; d Department of Animal Sciences, Shiraz University, Shiraz, Iran

**Keywords:** Cerebrospinal fluid, Hair follicle stem cells, Neuron, Glia

## Abstract

Hair follicle stem cells (HFSCs), owing to their accessibility and neural-like differentiation potential, represent a promising model for studying stem cell responses to central nervous system (CNS) microenvironments. Cerebrospinal fluid (CSF), a dynamic component of the CNS, contains stage-dependent bioactive factors that reflect developmental and functional states; however, their effects on peripheral stem cells remain unclear. This study investigated how CSF from distinct developmental stages modulates the viability, cellular metabolic activity, morphology, and neural-associated gene expression of rat HFSCs. HFSCs isolated from rat whisker pad skin were cultured in medium supplemented with 20% CSF collected from postnatal day 5 (P5) or adult rats and compared with cells maintained in 20% fetal bovine serum (FBS). Cell viability, cellular metabolic activity, neurite-like morphology, and gene expression were evaluated using FDA staining, MTT assay, crystal violet staining, and qRT-PCR, respectively. P5 CSF maintained HFSC viability and cellular metabolic activity at levels comparable to the FBS control and supported neurite-like extension. In contrast, adult CSF significantly reduced cellular metabolic activity and neurite-like extension length. Gene expression analysis demonstrated increased β-III Tubulin and PDGFR-α expression in P5 CSF-treated cells, whereas adult CSF upregulated MAP2 and Nestin expression. In addition, doublecortin, synapsin, and PSD-95 exhibited an increasing trend in adult CSF-treated cells, with synapsin showing significant elevation. These findings demonstrate that CSF exerts developmental stage–specific effects on HFSC behavior, with P5 CSF supporting cellular viability, metabolic activity, and neural-associated features, while adult CSF favors markers associated with neuronal maturation. Collectively, these findings highlight the potential utility of HFSCs as an accessible in vitro model for investigating how physiological and pathological changes in CSF composition influence stem cell behavior and neural-associated properties.

## Highlights

• Cerebrospinal fluid exerts developmental stage-dependent effects on hair follicle stem cells.
• P5 CSF maintained HFSC viability and metabolic activity and preserved neurite-like extensions.
• Adult CSF reduced HFSC viability, metabolic activity, and neurite-like extension length.
• P5 and adult CSF differentially regulated neural-associated gene expression.
• HFSCs provide a responsive in vitro model for investigating developmental stage-dependent CSF effects.

## Graphical Abstract

**Figure:**
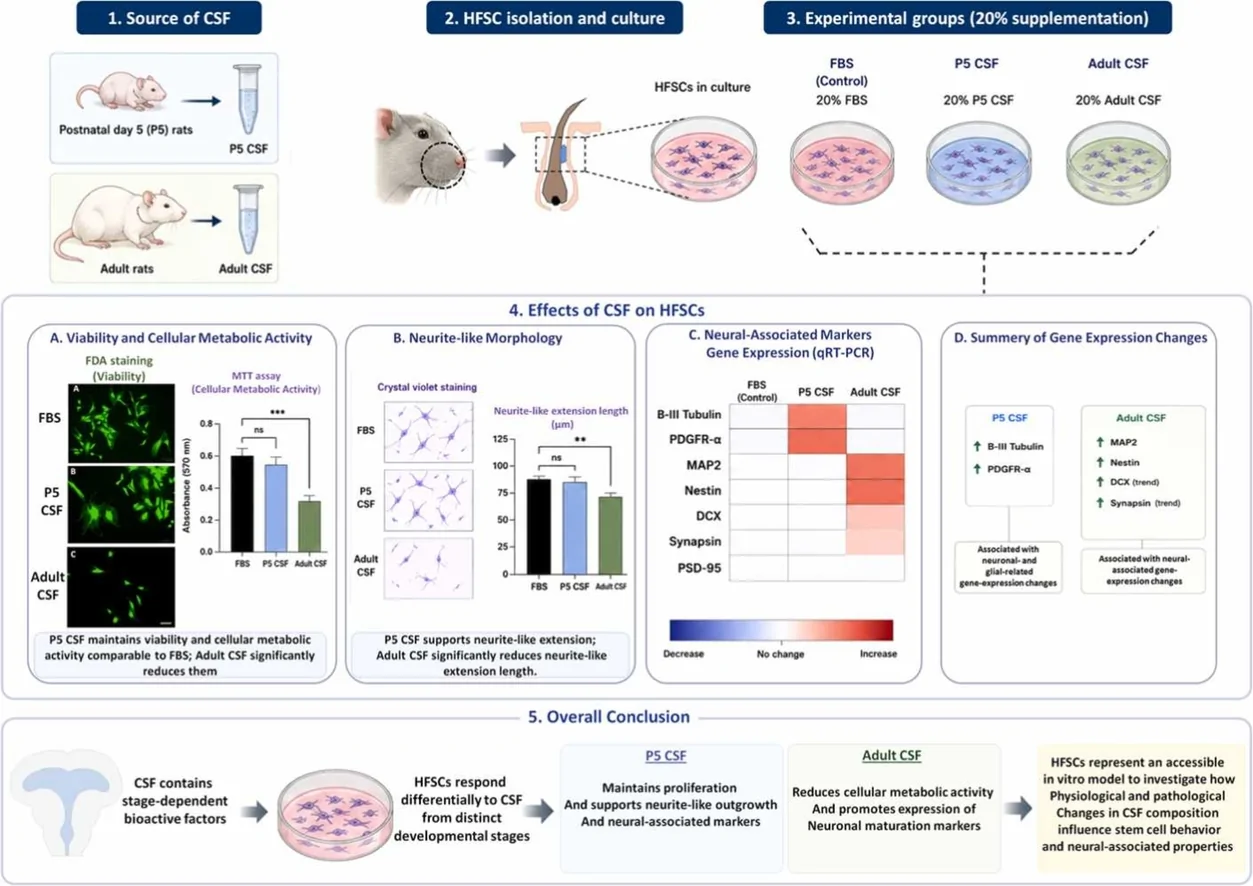

## Introduction

Neurogenesis and gliogenesis, the processes by which neural stem and progenitor cells generate neurons and glial cells, are fundamental for central nervous system (CNS) development, maintenance, and repair. Although these processes predominantly occur during embryonic and early postnatal development, neural stem cells retain a limited capacity for self-renewal and neural-associated plasticity throughout adulthood under appropriate microenvironmental conditions (Ming and Song, 2011). Understanding the extracellular signals that regulate these processes remains a major objective in neuroscience, particularly in the context of neurodevelopmental research and regenerative medicine. Hair follicle stem cells (HFSCs), located within the bulge region of adult hair follicles, have emerged as an accessible and multipotent stem cell population capable of exhibiting neural-like characteristics under specific environmental conditions (Sieber-Blum and Hu, 2008, Amoh et al., 2005a, Wu et al., 2017, Khodabakhsh et al., 2021). Owing to their accessibility, proliferative capacity, and responsiveness to neural-associated cues, HFSCs represent a promising in vitro model for investigating factors that influence stem cell behavior and neural-associated plasticity (Pandamooz et al., 2016). Cerebrospinal fluid (CSF) is a dynamic component of the CNS microenvironment that contains growth factors, cytokines, neurotransmitters, and other signaling molecules involved in regulating neural stem cell proliferation, differentiation, and maturation (Lehtinen and Walsh, 2011, Zappaterra and Lehtinen, 2012, Pandamooz et al., 2022). Importantly, CSF composition changes across developmental stages, reflecting the evolving physiological requirements of the developing and mature brain (Fame and Lehtinen, 2020, Gato et al., 2020). During embryonic and early postnatal development, CSF is enriched with neurotrophic and growth-promoting factors that support neurogenesis, gliogenesis, and neural progenitor survival (Lehtinen et al., 2011). In addition to sustaining cell viability, CSF has been implicated in regulating neurite outgrowth, synaptogenesis, and neural network formation (Kaneko and Sawamoto, 2018, Miyan et al., 2006). These observations highlight the important role of CSF-derived extracellular cues in shaping stem cell behavior during CNS development and maintenance. Despite increasing interest in CSF-mediated signaling, the influence of early postnatal CSF on peripheral stem cell behavior remains poorly understood. Furthermore, many studies investigating CSF effects have focused on neural stem cells that are difficult to isolate and maintain in vitro, or stem cells that do not fully mimic the behavior of neural stem cells, limiting their translational applicability (Radoszkiewicz et al., 2024, Ren et al., 2018).

In the present study, we investigated the effects of CSF collected from postnatal day 5 (P5) and adult rats on HFSC viability, metabolic activity, neurite-like morphology, and neural-associated gene expression. Postnatal day 5 (P5) was selected as the representative early postnatal developmental stage because it falls within an active period of postnatal neural development in the rat, characterized by ongoing neural progenitor proliferation, migration, differentiation, and glial development. Previous studies have demonstrated substantial cellular remodeling and continued neurogenic and gliogenic activity in the neonatal rat brain during this period (Staugaitis et al., 2001, Levison and Goldman, 1993, Levison et al., 1993, Luskin, 1993, Bandeira et al., 2009). Therefore, P5 CSF was selected to represent a developmentally active early postnatal CSF environment, whereas CSF from 3 to 4-month-old adult rats was used to represent the mature CSF environment.

By examining markers associated with neuronal, glial, progenitor, and synaptic development, we aimed to determine whether developmental stage–specific CSF environments differentially influence HFSC behavior and neural-associated plasticity. Collectively, this study may provide insight into how CSF-derived extracellular signals regulate stem cell behavior and support the potential use of HFSCs as an accessible in vitro model for studying CNS microenvironmental signaling under physiological and pathological conditions.

## Materials and methods

Animal care and experimental procedures were conducted in accordance with the guidelines set by the Animal Care Committee of Shiraz University of Medical Sciences and complied with internationally recognized ARRIVE guidelines. Ethical approval for this study was granted by the Research Ethics Committees of Laboratory Animals at Shiraz University of Medical Sciences (Approval ID: IR.SUMS.AEC.1402.002).

### Cerebrospinal fluid collection

#### Postnatal CSF collection

Cerebrospinal fluid (CSF) was collected from postnatal day 5 (P5) Sprague Dawley rat pups, representing an early postnatal period characterized by active neural progenitor proliferation, migration, differentiation, and ongoing neuronal and glial development. Following rinsing with sterile phosphate-buffered saline (PBS), pups were positioned to expose the cisterna magna (CM). A custom-made collection system consisting of a fire-polished glass microcapillary connected to a 100 μL pipette tip was carefully inserted into the CM for CSF aspiration while avoiding contact with surrounding tissues (Fig. 1A), as previously described (Tavakkoli et al., 2023). Although CSF collection from P5 pups requires careful handling because of their small size, the anatomical characteristics of this developmental stage facilitate access to the cisterna magna. During each collection session, CSF collected from the pups originating from the same dam was pooled to generate one collection batch. CSF batches from different collection sessions were kept separate and stored at −80°C until use. Prior to each experiment, one or more frozen CSF batches from the P5 group were thawed and combined to prepare the P5 CSF treatment.

**Fig. 1:**
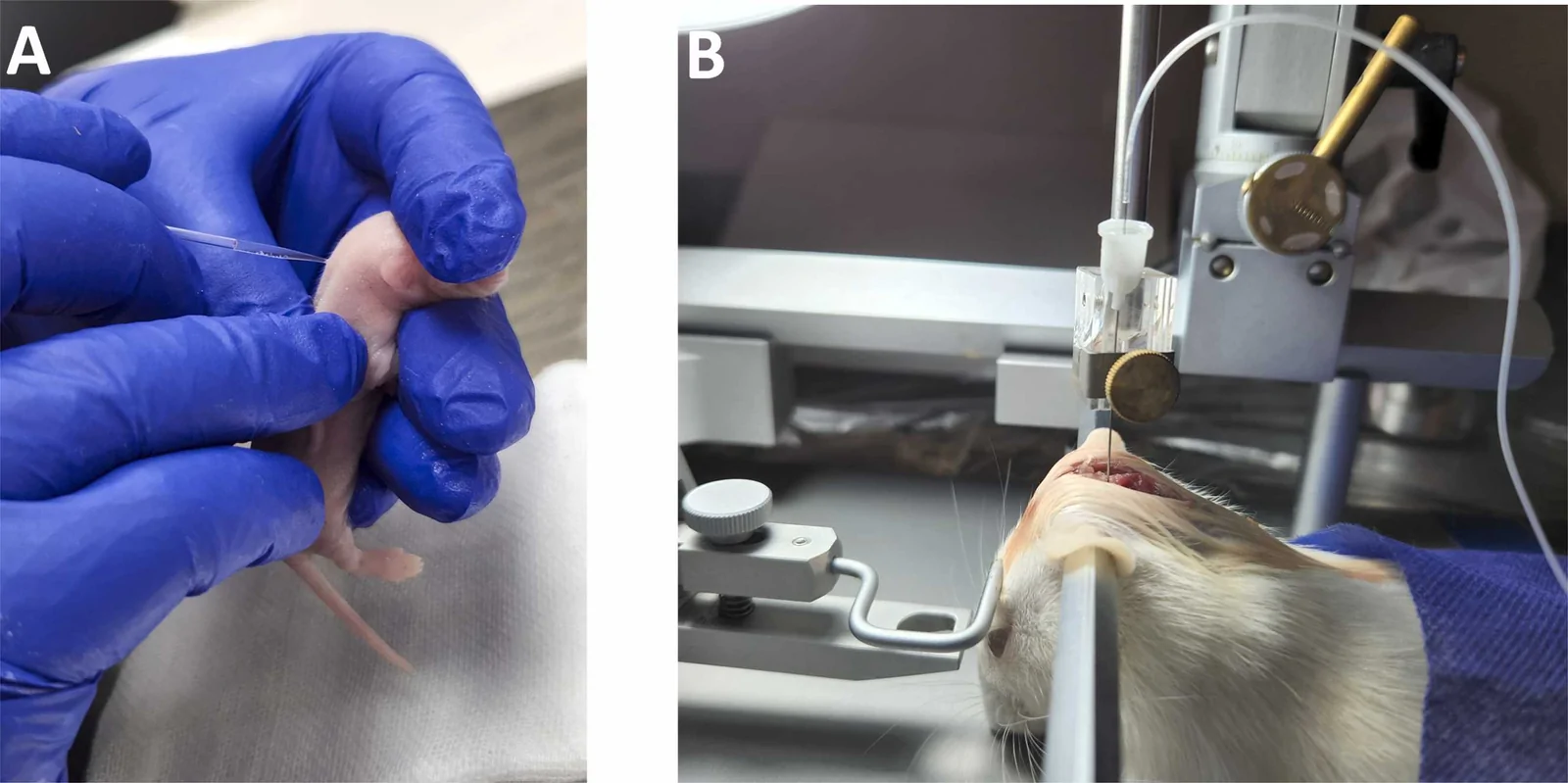
Cerebrospinal fluid collection from two developmental stages. CSF was collected from the cisterna magna of P5 pups using a fire-polished microcapillary pipette attached to a 100-μl tip (A). Adult CSF was collected from the cisterna magna of rats using a 27 G needle connected to a PE-10 tube, following animal fixation in a stereotaxic frame (B).

### Adult CSF collection

Adult CSF was collected from 3 to 4-month-old Sprague Dawley rats under anesthesia induced by ketamine (100 mg/kg; NARKAMON, Bioveta, Czech Republic) and xylazine (5 mg/kg; XYLASED, Bioveta, Czech Republic). Animals were positioned in a stereotaxic frame (TSE Systems, Germany), and CSF was collected from the cisterna magna using a 27 G dental needle connected to polyethylene-10 tubing and a 1 mL syringe, as previously described (Zarghami et al., 2013) (Fig. 1B).

Following collection, CSF samples from both age groups were transferred into sterile microtubes and centrifuged at 10,000*g* for 10 min at 4°C to remove cellular contaminants. Supernatants were collected and stored at −80°C until use. CSF samples collected during separate collection sessions were maintained as separate batches. Prior to each experiment, one or more CSF batches from the adult group were thawed and combined to prepare the adult CSF treatment.

### CSF analysis

The quality of CSF samples was assessed by protein quantification and cell counting. Protein concentration was determined using a bicinchoninic acid (BCA) assay. Detailed procedures have been described previously (Tavakkoli et al., 2023).

### Hair follicle stem cells isolation and culture

Isolation of hair follicle stem cells (HFSCs) were isolated from the bulge region of rat whisker follicles according to established protocols (Pandamooz et al., 2023). Briefly, whisker follicles were dissected, and bulge regions were delicately separated and plated onto collagen-coated 4-well culture plates. Explants were cultured in α-modified minimum essential medium (α-MEM; Bio-idea, Iran) supplemented with 10% fetal bovine serum (FBS; Bio-idea, Iran), 10% chick embryo extract (CEE), and 1% penicillin/streptomycin (P/S; Bio-idea, Iran). Cultures were maintained at 37°C in a humidified atmosphere containing 5% CO₂, and half of the medium was replaced every 2 days. After 7 days, migrated cells were dissociated by trypsinization and expanded for subsequent experiments.

### Immunofluorescent staining

Immunofluorescent staining was performed to confirm the identity of migrated stem cells at passage 1, as previously described (Pandamooz et al., 2023). Cells were incubated overnight at 4°C with mouse anti-nestin (1:50, Abcam, #ab6142) and rabbit anti-SOX-10 (1:100, Proteintech, #10422–1-AP) primary antibodies. After washing and blocking, cells were incubated with secondary antibodies—goat anti-mouse IgG AlexaFluor488 (1:1000, ThermoFisher, #A-11001) and goat anti-rabbit IgG AlexaFluor488 (1:1000, ThermoFisher, #A-11008)—for 2 h at room temperature. Hoechst 33342 (Sigma-Aldrich, #B2261) was used for nuclear counterstaining, and images were captured with a ZOE fluorescence microscope (Bio-Rad).

### Flow cytometry

The immunophenotypic profile of passage 3 HFSCs was assessed using flow cytometry with fluorochrome-conjugated antibodies against CD44 (CD44-FITC, BioLegend, #203906) and CD90 (CD90-PerCPCY5.5, BioLegend, #202515). At least 1 × 10⁵ cells were incubated with antibodies at 4°C for 30 min. Data acquisition was conducted on a BD FACSCalibur (BD Biosciences), and histogram analysis was performed using FlowJo v10 software.

### Treatment of HFSCs with CSF

To evaluate whether CSF from distinct developmental stages differentially modulates HFSC behavior, passage 3 cells were assigned to the following experimental groups. Experimental groups were delineated as follows: (i) Control Group (CTRL): HFSCs cultured in routine complete medium consisting of α-MEM supplemented with 20% FBS, 1% P/S, 1% L-Glu, and 1% NEAA, without CSF treatment. (ii) P5-CSF group: HFSCs cultured in α-MEM supplemented with 20% P5-CSF. (iii) Adult-CSF group: HFSCs cultured in α-MEM supplemented with 20% adult CSF. After 3 days of culture, cells were processed for subsequent analyses. For each experiment, one or more stored CSF batches from the same developmental stage were thawed and combined to prepare the stage-specific pooled CSF treatment. This approach provided sufficient CSF volume for the experiments while maintaining consistency of the CSF preparation within each experimental assay.

### Assessment of HFSC viability and cellular metabolic activity

Cell viability and cellular metabolic activity were evaluated using fluorescein diacetate (FDA) staining and the 3-(4,5-Dimethylthiazol-2-yl)-2,5-diphenyltetrazolium bromide (MTT) assay. HFSCs were treated with 20% CSF obtained from either P5 or adult rats for 72 h. Because of the limited availability of CSF, the number of wells used for each treatment group was restricted. For FDA staining, HFSCs were seeded at a density of 1.2 × 10⁴ cells/well in 12-well plates. Three wells were used for the control group, three wells for the P5-CSF group, and three wells for the adult-CSF group. Following 72 h of treatment, cells were incubated with FDA solution (25 μg/mL; Sigma-Aldrich, #F7378) for 5 min and imaged using a ZOE fluorescence microscope. Two fields of view were captured from each well, and the number of viable cells was quantified in each field. For the MTT assay, HFSCs were seeded in 96-well plates at density of 10^4^ cells/well and treated with 20% P5 or adult CSF for 72 h. Eight wells were used for the control group, six wells for the P5-CSF group, and six wells for the adult-CSF group. Cell metabolic activity was assessed using MTT (Sigma-Aldrich, #M5655) according to our previously described protocol (Pandamooz et al., 2024a). Absorbance was measured at 570 nm using a BioTek microplate reader.

### Neurite-like extension measurement

To assess CSF-induced morphological changes and neurite-like extension formation, cells were seeded in 12-well plates at a density of 1.2 × 10⁴ cells/well. After 24 h, cells were treated with 20% P5-CSF or adult CSF for an additional 24 h. Three wells were used for each group (control, P5-CSF, and adult CSF). Cells were then fixed with 4% paraformaldehyde for 15 min, washed with PBS, and stained with 0.5% crystal violet. Five images were acquired from each well, resulting in 15 images per experimental group. The length of cellular extensions was quantified using ImageJ (Fiji version 1.52r). The longest clearly identifiable neurite-like extension of each cell was manually traced and measured from the edge of the nucleus to the distal tip of the projection. Only cells with clearly identifiable cellular extensions and fully visible cell bodies were included in the analysis. Cells exhibiting extensions in contact with neighboring cells were excluded from measurement. Individual cell measurements were plotted to show the distribution of neurite-like extension lengths within each experimental group.

### RNA extraction, cDNA synthesis, and qRT‑PCR

For gene expression analysis, HFSCs were seeded at a density of 1.6 × 10⁵ cells/well in 6-well plates and allowed to attach for 24 h. In each experimental run, one well assigned to each experimental group. Cells were treated with 20% P5 or adult CSF for 72 h treatment period. Total RNA was extracted from each experimental well using a commercial RNA extraction kit (Cat. No: YT9063; Yekta Tajhiz Azma, Iran). Complementary DNA (cDNA) synthesis was performed using a reverse transcriptase kit (Cat. No: YT4500; Yekta Tajhiz Azma). Quantitative real-time PCR (qRT-PCR) was performed using gene-specific primers (Table 1) and RealQ Plus 2x Master Mix Green (Cat. No: A325402; Ampliqon, Denmark). Each cDNA sample was analyzed in technical triplicate. The Hprt gene served as the internal control, and relative expression levels were calculated using the 2^−^ΔΔCt^ method.

**Table 1:** List of primers.

Gene	Forward primer (5 ´-3 ´)	Reverse primer (5 ´-3 ´)
*Nestin*	CAAGGTCTGGTCTGGTGTATGC	GCTTTATTCAGGGAGGAAGAGAGG
*Dcx*	CGCCGCAGCAAGTCTCCAG	TCGCCAAGTGAATCAGAGTCATCC
*Β-III tubulin*	GCTGGAACGCATCAGTGTCTAC	GCACCACTCTGACCGAAGATAAAG
*Map2*	AAGCGGAAAACCACAGCAACAAG	TTCTCCTCCCTGTCTCCTGATACG
*Pdgfr-α*	AACCGAGGAGAACAACAGTAGCC	AAGAATCCGTCATGCCGAGAGG
*Psd-95*	TCACAGAGTGCTTCTCAGCCATCG	AGCCAGGGCAGGAATCAGAGTC
*Synapsin*	GACGGAGAGTGCCCTCAACATC	CAAACACAGCCACGGTGACAAAG
*hprt*	CCAGCGTCGTGATTAGTGATGATG	GAGCAAGTCTTTCAGTCCTGTCC

### Statistical analysis

Statistical analyses were performed using GraphPad Prism (Version 9, GraphPad Software Inc., San Diego, CA). Data are expressed as mean ± standard error of the mean (SEM). The Shapiro–Wilk test was used to assess normality. Group comparisons were conducted using one-way analysis of variance (ANOVA) followed by Tukey’s post hoc test. A significance level of P < 0.05 was considered statistically significant. All experiments were performed in triplicate.

## Results

### Migrated hair follicle stem cells and their characterization

Hair follicles were isolated from rat whisker pads, and the bulge region was dissected and cultured under defined conditions (Fig. 2A). Migrating cells were observed surrounding the explanted bulge regions within 2–3 days of culture (Fig. 2B).

**Fig. 2:**
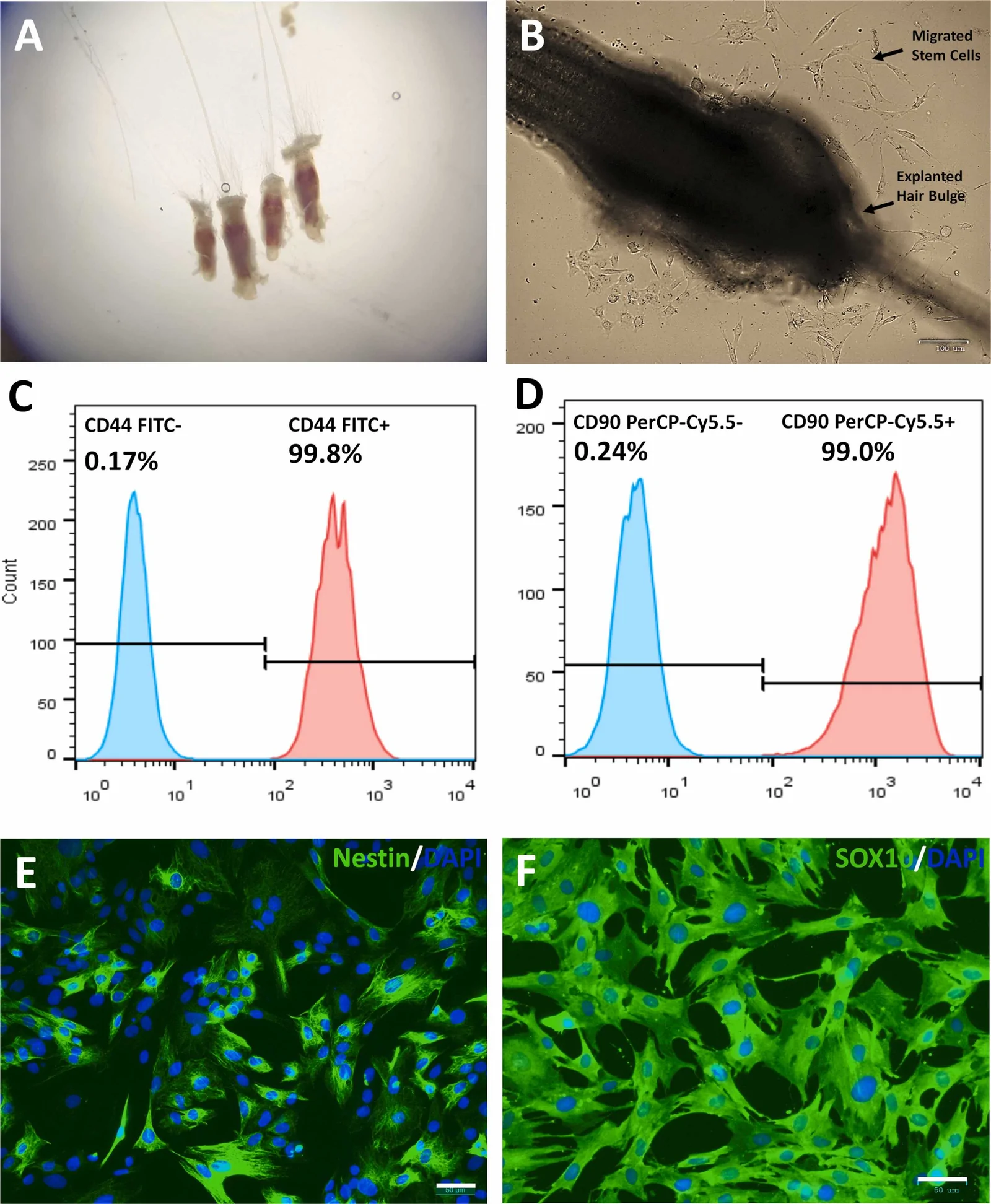
Isolation and characterization of rat hair follicle stem cells. Hair follicles were dissected from the whisker pad of rats (A). Two to three days post-explantation, migrated stem cells were observed around the bulge region, scale bar: 100 µm (B). Flow cytometry analysis of key cell surface markers at passage three revealed robust expression of CD44 and CD90 (C, D). Immunostaining against Nestin and SOX10 following the first subculture confirmed the identity of migrated stem cells as HFSCs, scale bar: 50 µm (E, F). Cell nuclei were counterstained with Hoechst 33342 dye. The images provided represent examples of 3 distinct assessments for each immunostaining (n = 3).

Flow cytometric analysis of passage 3 cells demonstrated high expression of the mesenchymal stem cell markers CD44 and CD90, with positivity rates of 99.8% and 99.0%, respectively (Fig. 2C, D). These markers are commonly associated with multipotent mesenchymal/stromal stem cell populations, as extensively documented (Vasyliev et al., 2019).

In addition, immunofluorescence staining at passage 1 confirmed the expression of Nestin and SOX-10 in migrated cells (Fig. 2E, F), supporting their characterization as HFSCs with neural crest-associated features. Nestin, a well-established marker of neural crest-derived cells, is known to be expressed in hair follicle stem cell populations (Amoh et al., 2005b, Baba et al., 2022). Moreover, SOX-10, a transcription factor essential for neural crest development, has been reported as a highly sensitive marker for the immunodetection of rat neural crest-derived stem cells (Yang et al., 2020, Jing et al., 2021).

### Analysis of CSF from adult and postnatal stages

The average volume of CSF collected from each adult rat ranged from 100 to 200 μl, with a mean protein concentration of 341.98 µg/mL. In contrast, CSF collected from postnatal day 5 (P5) rats yielded an average volume of 20–30 μL and exhibited a higher protein concentration of 1777.33 μg/mL, with an overall collection success rate of 96%.

### The influence of postnatal and adult CSF on HFSC viability and metabolic activity

Cell viability was assessed using fluorescein diacetate (FDA) staining, which demonstrated differences in the number of viable cells across experimental groups. Quantitative image analysis revealed a significantly lower number of live cells in the adult CSF-treated group compared with the control group (P < 0.001) (Fig. 3A–D). In parallel, the MTT assay was used to assess cellular metabolic activity following CSF treatment. HFSCs treated with P5 CSF exhibited MTT activity comparable to that of the FBS-supplemented control group (P = 0.37), whereas adult CSF treatment resulted in a significant reduction in MTT activity (P < 0.001) (Fig. 3E). Collectively, both assays indicate that CSF from different developmental stages differentially affects HFSC viability and cellular metabolic activity. P5 CSF maintained cellular activity comparable to control conditions, whereas adult CSF was associated with reduced viability and metabolic activity. Because MTT activity can be influenced by cellular metabolic state and does not directly measure cell proliferation, these findings were interpreted as changes in cellular metabolic activity rather than as direct evidence of altered proliferation.

**Fig. 3:**
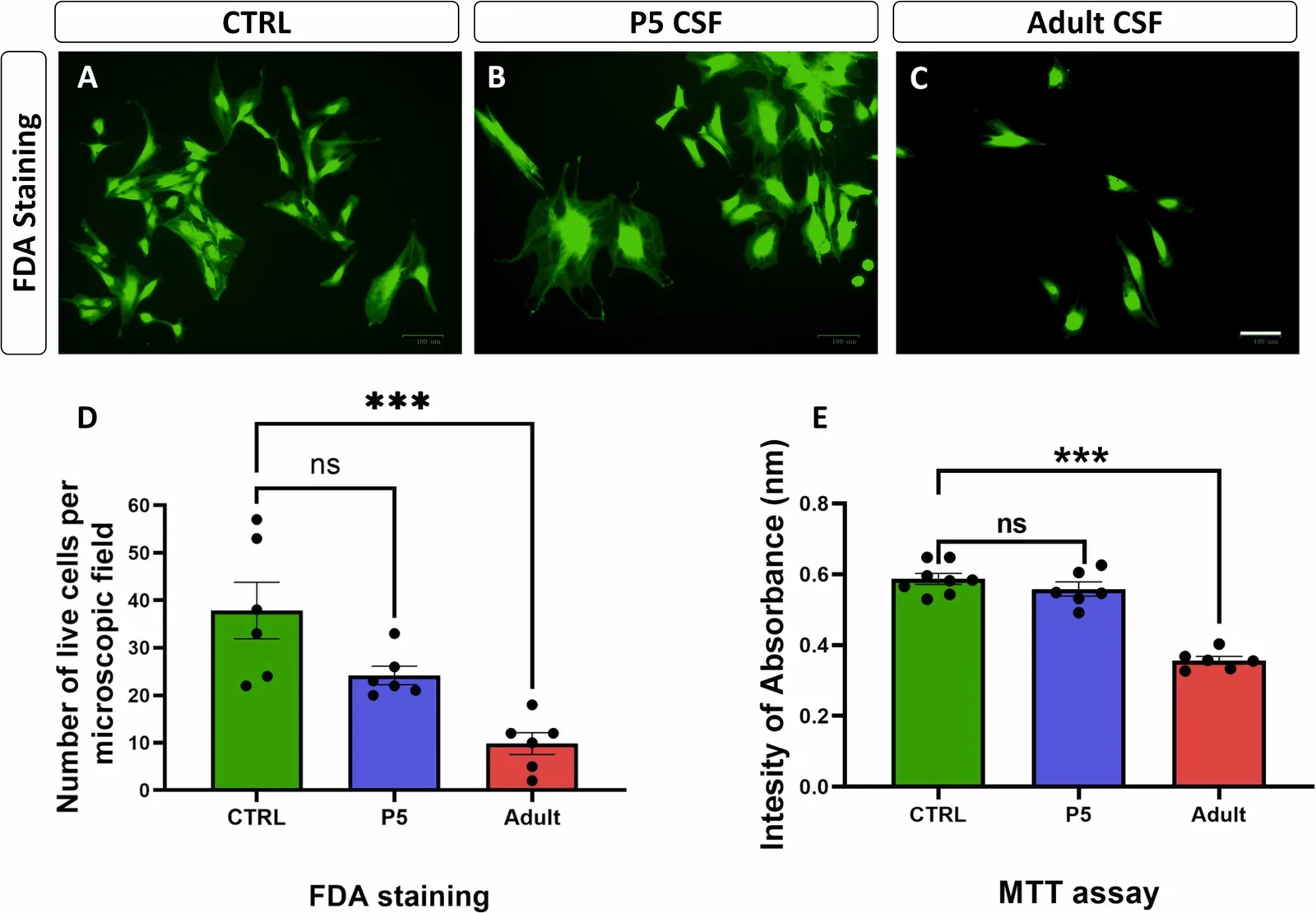
Evaluation of the impact of P5 and adult CSF on HFSCs viability and metabolic activity. FDA staining of stem cells post-treatment with CSF from both developmental stages showed the number of live cells in each experimental group (A-C). Images were randomly captured from three wells per experimental group, with representative images selected from three separate experiments. Scale bar: 50 µm. Statistical analysis revealed a significantly lower number of live stem cells per microscopic field in the group treated with adult CSF compared to the control group (P < 0.001) (D). MTT assay performed after 72 h of treatment demonstrated comparable cellular metabolic activity between the P5 CSF-treated and control groups (P = 0.37), whereas adult CSF treatment significantly reduced MTT activity compared with the control group (P < 0.001) (E).

### Effect of postnatal 5 and adult CSF on neurite-like extensions of HFSCs

Analysis of neurite-like extensions length in HFSCs following treatment with P5 and adult CSF revealed distinct effects across experimental groups. HFSCs treated with P5 CSF exhibited an average neurite-like extension length of 90 ± 2.83 µm, which was not significantly different from the control group (89.09 ± 3.37 µm; P = 0.96). In contrast, HFSCs exposed to adult CSF exhibited a significantly shorter mean neurite-like extension length (76.74 ± 2.36 µm) compared with control cells (P = 0.008) (Fig. 4). These findings indicate that P5 CSF does not significantly alter neurite-like extension length relative to control conditions, whereas adult CSF was associated with a reduction in the length of these cellular extensions.

**Fig. 4:**
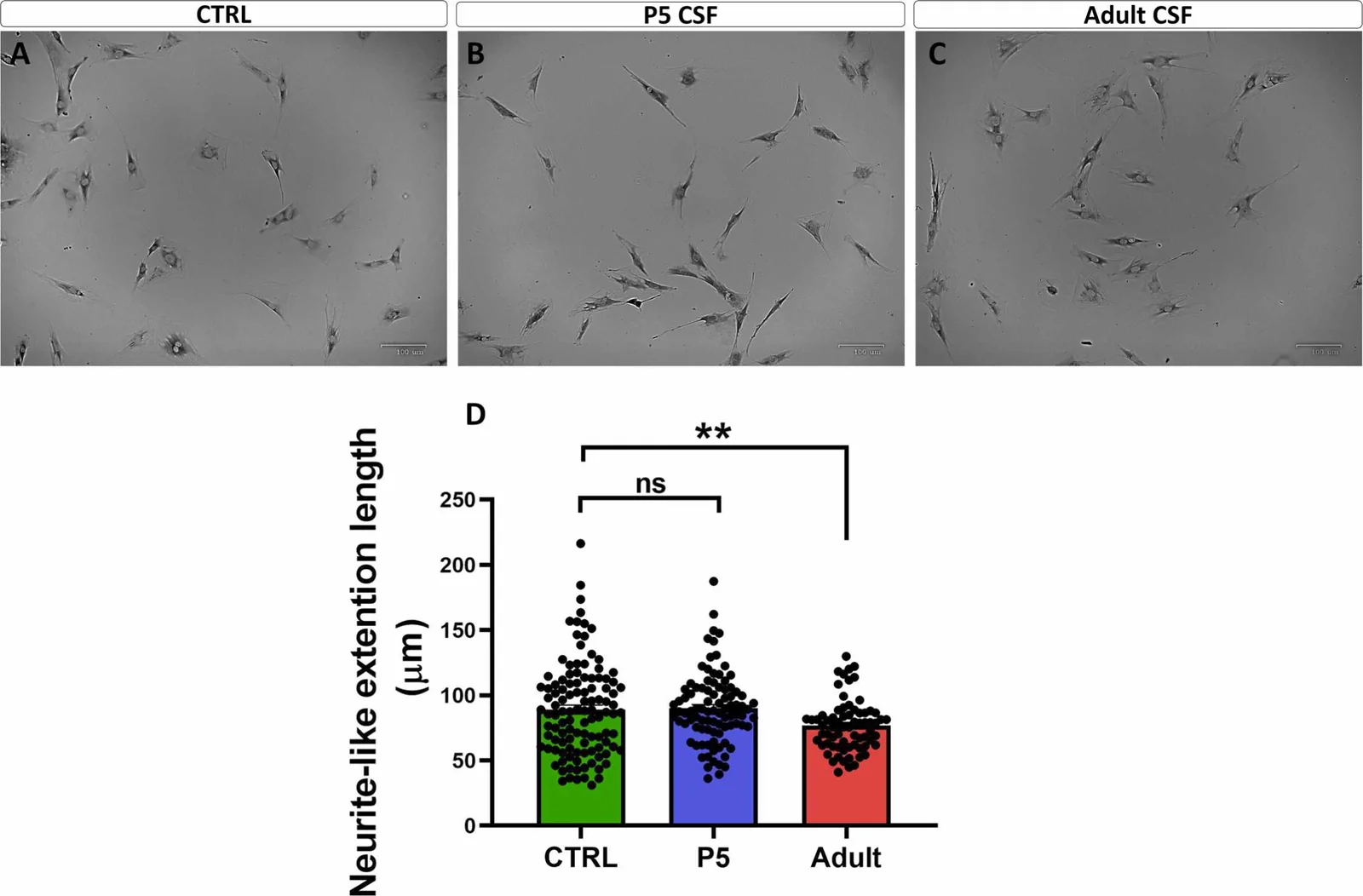
Assessment of the impact of P5 and adult CSF on HFSCs neurite-like extensions. Crystal violet staining was used to visualize cellular morphology and neurite-like extensions in HFSCs across different treatment groups (A-C). In control group (supplemented with 20% FBS), stem cells displayed neurite-like extensions with a mean length of 89.09 ± 3.37 µm. P5-CSF-treated cells exhibited a mean extension length of 90.00 ± 2.83 µm, with no significant difference compared with the control group (P = 0.96). (P = 0.96). Adult CSF treatment resulted in a significantly shorter mean neurite-like extension length (76.74 ± 2.36 µm) compared with the control group (P = 0.008) (D). Data are presented as mean ± SEM. Statistical differences between groups were determined using one-way ANOVA. **P < 0.01 compared to the control group. Scale bar = 100 µm.

### The effect of postnatal Day 5 and adult CSF on the expression of neural-associated markers

To investigate the influence of CSF on HFSC neural-associated gene expression, quantitative real-time PCR (qRT-PCR) was performed to evaluate the expression of neuronal, glial, progenitor, and synaptic markers. Expression of the early neuronal marker β-III Tubulin was significantly increased in P5 CSF-treated cells compared to the control group (P < 0.01), whereas adult CSF did not induce a significant change (P = 0.69). In contrast, expression of microtubule-associated protein 2 (MAP2), a marker associated with neuronal maturation, was significantly elevated in the adult CSF-treated group compared with controls (P < 0.05), while P5 CSF treatment resulted in a non-significant reduction in MAP2 expression (P = 0.15).

To further assess neural-associated lineage responses, the expression of platelet-derived growth factor receptor alpha (PDGFRα), a marker associated with oligodendrocyte precursor cells, was examined. P5 CSF treatment significantly increased PDGFRα expression compared with the control group (p < 0.01), whereas adult CSF did not induce a comparable effect.

Nestin expression was also evaluated as a marker associated with neural stem/progenitor characteristics. HFSCs treated with adult CSF exhibited a marked increase in Nestin expression relative to the control group (P < 0.0001), while no significant difference was observed between the P5 CSF-treated and control groups (P = 0.48).

Additionally, we assessed the expression of doublecortin (DCX), a marker associated with neuronal precursor cells and immature neurons, was evaluated following CSF treatment. No significant changes in DCX expression were observed in either the P5 CSF-treated group (P = 0.97) or the adult CSF-treated group (P = 0.12) compared with the control group, although the adult CSF-treated cells showed an increasing trend in expression.

Finally, expression levels of the synaptic-associated markers PSD-95 and Synapsin were evaluated. Although PSD-95 expression was detected in all experimental groups, no significant differences were observed between groups (P = 0.70 for P5 CSF vs control; P = 0.97 for adult CSF vs control). Similarly, Synapsin expression showed a decreasing trend in the P5 CSF-treated group compared with the control group, although this difference did not reach statistical significance (P = 0.05). In the adult CSF-treated group, Synapsin expression showed a slight increase; however, this change was also not statistically significant compared with controls (P = 0.73). Overall, these findings demonstrate that CSF from different developmental stages differentially modulates neural-associated gene expression in HFSCs (Fig. 5).

**Fig. 5:**
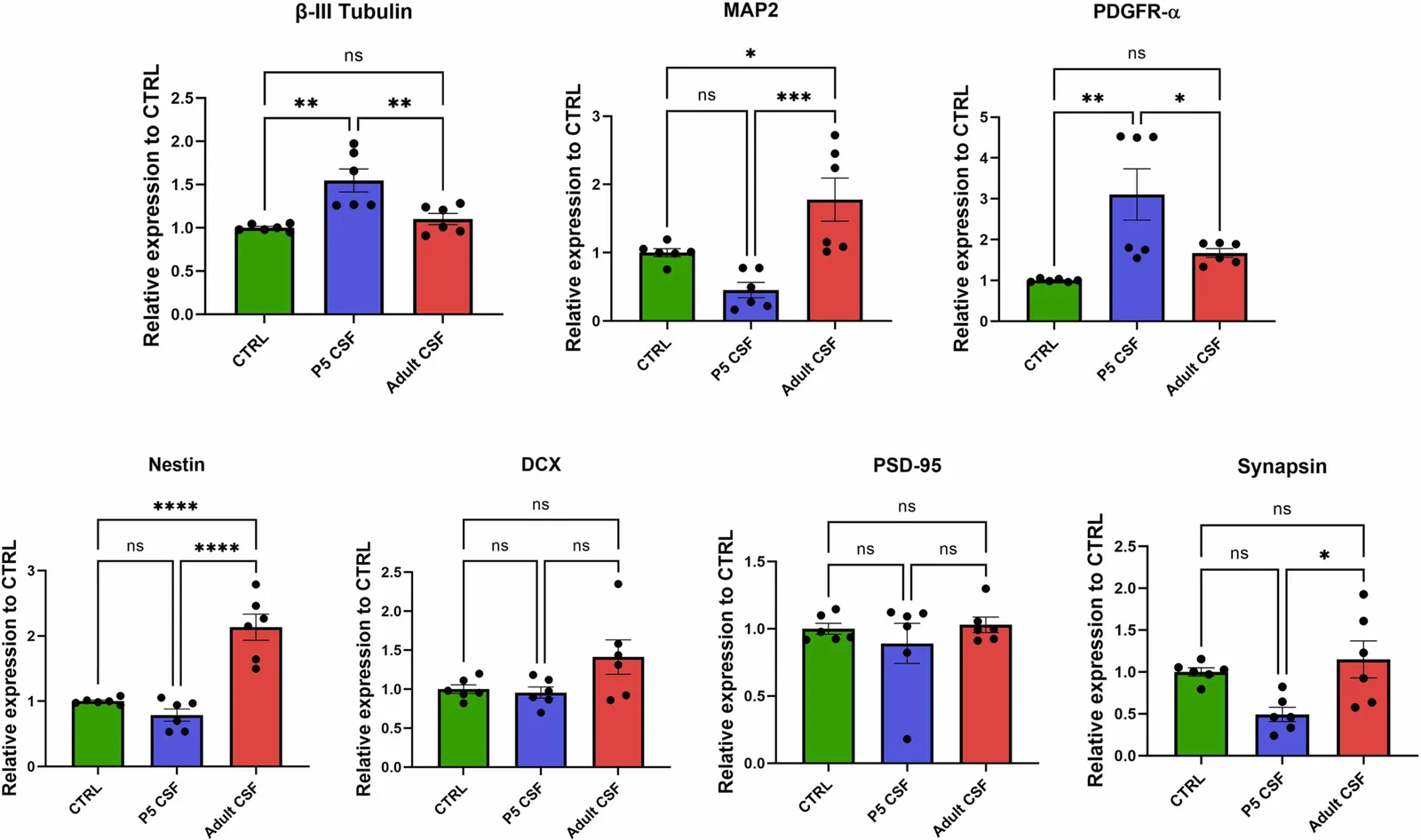
Gene expression analysis in HFSCs following CSF exposure. Quantitative real-time PCR analysis was used to evaluate major neuronal and glial markers in HFSCs following 72 h treatment with growth medium supplemented with either 20% P5 or adult CSF. The expression of β-III Tubulin, as an early neuronal marker, significantly increased in P5-CSF group compared to CTRL group supplemented with 20% FBS (P < 0.01).The expression of MAP2, a mature neuronal marker, was significantly enhanced in the adult CSF group (P < 0.05). The evaluation of PDGFRα, a glial and proliferation marker revealed a significant increase in P5-CSF treated cells (P < 0.01). Additionally, the expression of Nestin, a marker for neural stem/progenitor cells already expressed in HFSCs, significantly increased following adult CSF supplementation (P < 0.0001). Meanwhile, no significant changes were detected in the expression of DCX (marker for neuronal precursor cells and immature neurons), PSD-95 (marker for the formation of connections), and synapsin (marker for synaptogenesis). Data are presented as mean ± SEM. Statistical differences between groups were determined using one-way ANOVA, with a sample size of n = 6. Significance levels are denoted as *P < 0.05, **P < 0.01 and ****P < 0.0001 compared to the control group.

## Discussion

In the present study, we investigated the effects of cerebrospinal fluid (CSF) obtained from two developmental stages on the behavior and neural-associated gene expression of rat hair follicle stem cells (HFSCs). Our findings demonstrated that P5 CSF maintained HFSC viability, metabolic activity, and neurite-like morphology at levels comparable to the FBS control while increasing the expression of β-III Tubulin and PDGFRα. In contrast, adult CSF reduced viability, metabolic activity, and neurite-like extension while upregulating MAP2 and Nestin expression. Collectively, these findings indicate that developmental stage–specific CSF environments differentially influence HFSC behavior and neural-associated transcriptional profiles.

Although the composition and biological functions of fetal and adult CSF have been extensively investigated, relatively limited attention has been given to early postnatal CSF. Although other postnatal ages, including later neonatal stages, could also provide informative developmental CSF profiles, P5 was selected as a representative early postnatal time point to capture a developmentally active CSF environment with elevated levels of neurodevelopmentally relevant signaling molecules (Murtazina et al., 2021) while limiting the number of animals required for collection. This design allowed us to compare a single defined early postnatal stage with the adult state without introducing additional animal cohorts.

Previous studies examining developmental-stage CSF have demonstrated that neonatal or early postnatal CSF can support stem cell survival, proliferation, and neural-associated properties in various stem cell populations (Haratizadeh et al., 2017, Shokohi et al., 2017). Consistent with these observations, our findings suggest that P5 CSF provides a supportive microenvironment for HFSC viability, metabolic activity, and maintenance of neurite-like morphology. During early postnatal development, CSF is enriched with growth factors, cytokines, and neurotrophic molecules that are essential for rapid CNS growth and maturation (Lehtinen et al., 2011). The increased expression of β-III Tubulin and PDGFRα observed following P5 CSF treatment further supports the notion that early postnatal CSF contains bioactive factors capable of modulating neural- and glial-associated gene expression in HFSCs.

In contrast, HFSCs exposed to adult CSF exhibited reduced viability, metabolic activity, and neurite-like extension compared with control conditions. Consistent with these findings, our previous study demonstrated that culturing HFSCs solely in adult rat CSF collected from the CM maintained cell viability at levels comparable to serum-free α-MEM medium, although viability remained lower than in growth factor–supplemented conditions (Pandamooz et al., 2013). These findings further support the notion that adult CSF does not provide a highly proliferative environment for HFSCs.

Most previous studies investigating the effects of adult CSF have focused on human CSF, typically collected through lumbar puncture, and its influence on neural stem cells (NSCs) or neural progenitor populations. Several studies have reported that adult human CSF supports stem cell survival while reducing proliferation and promoting glial-associated or maturation-related properties (Buddensiek et al., 2009, Buddensiek et al., 2010, Ma et al., 2013). For example, Buddensiek et al. demonstrated that adult human CSF enhanced survival but inhibited proliferation and neuronal specification of fetal NSCs (Buddensiek et al., 2009). Similarly, Ma et al. reported that adult human CSF did not promote neuronal differentiation or survival of rat NSCs (Ma et al., 2013). Kiiski and colleagues further demonstrated that human CSF promoted glial differentiation in human embryonic stem cell-derived neural cultures while maintaining spontaneous neuronal network activity (Kiiski et al., 2013).

In contrast, other studies have suggested that adult CSF may preserve neuronal viability and support aspects of neuronal maturation under specific experimental conditions. Perez-Alcazar et al. showed that human CSF maintained neuronal morphology and synaptic activity in rat hippocampal slices (prepared from P7–10) and primary neuronal cultures (obtained from P0–3 rats) (Perez-Alcazar et al., 2016).

Similarly, studies using organotypic brain slices and induced pluripotent stem cell-derived neural cultures reported enhanced neuronal survival, increased MAP2 expression, and elevated synaptic-associated markers following exposure to human CSF (Schwarz et al., 2017, Izsak et al., 2020). Collectively, these findings suggest that adult CSF may primarily support cellular maintenance and maturation-related processes rather than highly proliferative states. Importantly, most previous studies have evaluated lumbar human CSF, which differs from ventricular CSF in protein composition and molecular content (Rostgaard et al., 2023).

In addition, many investigations utilized fetal-derived NSCs or induced pluripotent stem cell-derived neural populations, often without species or developmental-stage matching between donor CSF and target cells. In contrast, the present study employed rat cisterna magna-derived CSF together with rat HFSCs, providing a more developmentally and physiologically relevant model for investigating CSF-mediated effects on stem cell behavior.

In contrast, the present study utilized rat HFSCs, which possess neural crest-associated characteristics and the capacity to express neuronal and glial markers under appropriate experimental conditions (Pandamooz et al., 2023, Pandamooz et al., 2024b). These properties make HFSCs a relevant and accessible in vitro model for investigating the influence of CSF on stem cells that share developmental and physiological features with the CNS. This approach may provide a more representative model for studying adult stem cell responses to CNS-derived extracellular signals and may enhance the translational relevance of CSF-related research.

Furthermore, our findings demonstrated that P5 CSF supported neurite-like extension in HFSCs, suggesting the presence of developmental stage–specific bioactive factors capable of modulating neural-associated cellular morphology. Neurite outgrowth is an important feature associated with neural development, connectivity, and cellular communication within the nervous system. The maintenance of neurite-like extensions in the P5 CSF-treated group therefore supports the concept that early postnatal CSF provides a more permissive microenvironment for neural-associated cellular responses. In contrast, adult CSF reduced neurite-like extension, which may reflect the distinct functional composition of mature CSF that primarily supports CNS homeostasis rather than active developmental growth processes.

Regarding neural-associated gene expression, treatment with P5 CSF resulted in the simultaneous upregulation of β-III Tubulin and PDGFRα, suggesting that early postnatal CSF contains molecular cues capable of modulating both neuronal- and glial-associated transcriptional pathways in HFSCs. These findings are consistent with the dynamic role of early developmental CSF in regulating cellular growth and differentiation-related signaling within the developing CNS. The developmental context of the early postnatal period further supports this interpretation. Previous studies have demonstrated that the postnatal rat subventricular zone contains progenitor populations with the capacity to generate multiple neural and glial lineages. In particular, postnatal SVZ progenitors contribute to both oligodendrocyte and astrocyte populations, and their migratory patterns and developmental fates are temporally regulated during postnatal development (Levison and Goldman, 1993, Levison et al., 1993). Early astrocyte differentiation and cellular heterogeneity within the neonatal SVZ have also been reported (Staugaitis et al., 2001), while postnatally generated neurons arising from the SVZ contribute to neuronal populations in the developing forebrain (Luskin, 1993). Moreover, substantial changes in neuronal and non-neuronal cell populations occur during postnatal rat brain growth (Bandeira et al., 2009). Taken together, these findings provide a developmental framework for our observation that P5 CSF alters both β-III Tubulin and PDGFRα expression in HFSCs, suggesting that factors present in early postnatal CSF may influence molecular programs associated with both neuronal and glial development.

In contrast, HFSCs treated with adult CSF exhibited significantly increased MAP2 expression, while β-III Tubulin expression remained unchanged. This pattern may indicate that adult CSF preferentially influences transcriptional programs associated with neuronal maturation rather than early neuronal commitment. In addition, the increased expression of Nestin following adult CSF treatment suggests that adult CSF may also support the maintenance of progenitor-associated cellular characteristics.

The differential expression of neural-associated genes following exposure to P5 and adult CSF should be interpreted at the transcript level and does not by itself establish corresponding protein expression, neuronal or glial differentiation, or definitive lineage commitment. The relatively short treatment period used in the present study was selected primarily to assess early CSF-induced cellular responses and morphological changes. Longer-term exposure, assessment of lineage-specific proteins, and functional differentiation assays would be required to determine whether the observed transcriptional changes progress toward stable neuronal or glial phenotypes. Nevertheless, previous studies have demonstrated that postnatal CSF contains developmental signals capable of influencing both neuronal and glial progenitor behavior. Thus, the distinct transcriptional responses observed following P5 versus adult CSF exposure are consistent with the concept that CSF provides developmentally regulated extracellular cues. Within this framework, HFSCs may provide a useful responsive cellular model for investigating the biological effects of CSF obtained at different developmental stages.

From a translational perspective, the findings of this study highlight the potential utility of HFSCs as an accessible in vitro model for investigating how CSF environments from different developmental stages influence stem cell behavior and neural-associated molecular responses. The differential effects observed following exposure to P5 and adult CSF further support the concept that CSF-derived extracellular signals can modulate stem cell responses in a developmentally dependent manner. Future studies aimed at identifying the molecular mechanisms underlying these effects, together with longer-term treatment and protein-level and functional differentiation analyses, may provide further insight into the relationship between CSF developmental stage and stem cell behavior, with potential relevance to CNS microenvironmental regulation and regenerative medicine.

## Conclusion

This study demonstrates that cerebrospinal fluid (CSF) from different developmental stages differentially influences HFSC viability, metabolic activity, neurite-like morphology, and neural-associated gene expression. P5 CSF promoted viability, metabolic activity, and neural-associated features, including maintenance of neurite-like extensions and increased expression of β-III Tubulin and PDGFRα, whereas adult CSF was associated with reduced viability, metabolic activity, and increased expression of MAP2 and Nestin. These findings suggest that developmental stage–specific CSF environments differentially modulate the molecular and morphological characteristics of HFSCs. Importantly, the observed changes in neural-associated gene expression and cellular morphology do not by themselves establish definitive neuronal or glial differentiation. Nevertheless, the findings support the potential utility of HFSCs as an accessible in vitro model for investigating developmentally regulated CSF signaling and its effects on stem cell behavior. Further studies incorporating longer-term treatment, protein-level validation, and functional differentiation analyses are warranted to clarify the biological significance and mechanisms underlying these CSF-dependent effects.

## CRediT authorship contribution statement

**Sareh Pandamooz:** Writing – review & editing, Writing – original draft, Visualization, Validation, Resources, Project administration, Methodology, Investigation, Funding acquisition, Formal analysis, Data curation, Conceptualization. **Zahra Tavakkoli:** Writing – original draft, Investigation. **Jaleel A. Miyan:** Writing – review & editing, Validation, Supervision, Methodology, Conceptualization. **Mohammad Saied Salehi:** Writing – review & editing, Validation, Methodology, Investigation, Formal analysis, Conceptualization.

## Declaration of Generative AI and AI-assisted technologies in the writing process

During the preparation of this work the authors used ChatGPT in order to improve language and readability. After using this tool, the authors reviewed and edited the content as needed and takes full responsibility for the content of the published article.

## Ethics declaration

This study was conducted in accordance with the ARRIVE (Animal Research: Reporting of In Vivo Experiments) guidelines. This study was approved by the Shiraz University of Medical Sciences. (Approval No. IR.SUMS.AEC.1402.002)

### Compliance with ethical standards

Animal care and experimental procedures were conducted in accordance with the guidelines set by the Animal Care Committee of Shiraz University of Medical Sciences and complied with internationally recognized ARRIVE guidelines. Ethical approval for this study was granted by the Research Ethics Committees of Laboratory Animals at Shiraz University of Medical Sciences (Approval ID: IR.SUMS.AEC.1402.002).

## Funding

This study was financially supported by the IBRO Early Career Award 2023, granted to Sareh Pandamooz, and by 10.13039/501100004320Shiraz University of Medical Sciences (Grant No. 31242).

## Declaration of Competing Interest

The authors declare that they have no conflict of interest related to this study.

## Data Availability

The datasets generated and analyzed during the current study are available from the corresponding author upon request.
